# Factors influencing mortality in a captive breeding population of Loggerhead Shrike, Eastern subspecies (*Lanius ludovicianus* ssp.) in Canada

**DOI:** 10.1186/s12917-015-0429-2

**Published:** 2015-06-09

**Authors:** E. Jane Parmley, David L. Pearl, Nadine A. Vogt, Stephanie Yates, G. Douglas Campbell, Jessica Steiner, Tara L. Imlay, Simon Hollamby, Ken Tuininga, Ian K. Barker

**Affiliations:** Canadian Wildlife Health Cooperative – Ontario/Nunavut Region, Department of Pathobiology, University of Guelph, Guelph, Ontario N1G 2W1 Canada; Department of Population Medicine, University of Guelph, Guelph, Ontario N1G 2W1 Canada; Wildlife Preservation Canada, 5420 Highway 6 North, RR#5, Guelph, Ontario N1H 6J2 Canada; Biology Department, Dalhousie University, 1355 Oxford St., Halifax, Nova Scotia B3H 4R2 Canada; Toronto Zoo, 361A Old Finch Avenue, Toronto, Ontario M1B 5K7 Canada; Environment Canada, Canadian Wildlife Service - Ontario, 4905 Dufferin Street, Toronto, Ontario M3H 5T4 Canada

**Keywords:** Canada, Captive breeding, Eastern Loggerhead Shrike, *Lanius ludovicianus* ssp, Fledgling, Mortality, Survival

## Abstract

**Background:**

The Loggerhead Shrike, Eastern subspecies (*Lanius ludovicianus* ssp.) (LOSH) is a predatory songbird native to Eastern North America. It is estimated that there are fewer than 55 breeding pairs of this subspecies in North America. Captive breeding plays a critical role in preventing the extirpation of this subspecies from its Canadian range. Unfortunately, high numbers of unexplained deaths among young birds in the captive breeding population threatened the success of this program. This paper describes fledgling mortality in the captive breeding population, and seeks to identify factors associated with fledgling survival and, ultimately, to identify steps to mitigate fledgling mortality.

**Results:**

Over the study period (2006–2011) at two breeding sites, 696 LOSH were fledged. Among these, 68 % (n = 474) were released, 10 % (n = 69) were retained in the captive breeding population, and 22 % (n = 155) died. Fledgling survival declined from 99 % in 2006 to 44 % in 2011. The odds of survival were significantly lower for fledglings that were part of a second clutch. As the number of fledglings in a clutch increased, the odds of surviving increased significantly. As the breeding female aged from one to four years of age, there was a marked increase in the odds of a fledgling surviving, which then subsequently declined as females aged further.

**Conclusions:**

Based on our analyses, clutch number (first or second), number of fledglings in the brood, and age of breeding females were significant predictors of fledgling survival. Long-term breeding management decisions will have to balance the need to increase the number of individuals and breeding pairs in the wild by releasing large numbers of young, against the need to maintain a genetically viable captive population, until the wild population is large enough to be self-sustaining.

## Background

The Loggerhead Shrike, Eastern subspecies (*Lanius ludovicianus* ssp.) (LOSH) is a predatory songbird native to Eastern North America. They are highly territorial birds that breed in short grasslands where it is easier to find prey. Breeding pairs build their nests cooperatively and egg-laying starts in mid-late April in Ontario. Five to seven eggs are laid in a clutch and the incubation period is 16 days. Young LOSH fledge (become flighted) in 17–20 days and, as fledglings, continue to be fed by the parents for approximately 28 days more.

In 1991, LOSH was listed as endangered by the Committee on the Status of Endangered Wildlife in Canada (COSEWIC) [1]; it was also listed under the Species at Risk Act (SARA) as endangered in 2003 and is protected under the Migratory Birds Convention Act 1994 (MBCA) [2]. In May 2014, the subspecies *migrans* was de-activated and the Eastern Canada population was considered to represent an unnamed Eastern subspecies (*L. ludovicianus* ssp.), which remains listed as endangered [3]. Most of the Canadian population is believed to be limited to scattered small areas in Ontario, though it also breeds rarely in Québec [2]. It is estimated that there are fewer than 55 breeding pairs in North America and fewer than 110 individual birds [3]. The Eastern subspecies migrates to south-central United States, although the southern distribution of the wintering population remains uncertain. Young and adults alike begin their southward migration in early-mid August [1].

The cause of the population decline in Canada is not fully understood, but loss and fragmentation of breeding and wintering habitat is believed to be a major contributing factor [4]. Other hypotheses include environmental contaminants (e.g. pesticides), infectious disease, motor vehicle collisions, extreme weather events, predation and competition with other bird species that are more tolerant to changing environments [5–8].

A captive breeding program was started in 1997, with the following goals: 1) to recover the LOSH population in Ontario and Québec; and, 2) to preserve the genetic diversity of the wild population. The program was first established at the Toronto Zoo (Ontario) and McGill University (Québec) with the recruitment of wild nestlings to form a captive breeding population. In 2001, experimental in-situ captive breeding and release was initiated (see [9] for full description of this method).

The program is currently managed by Wildlife Preservation Canada (WPC) on behalf of Environment Canada, Canadian Wildlife Service (CWS) – Ontario. Between 2006 and 2012, over 500 captive-bred young were released into the wild. Once released, captive-reared shrikes have high survival [10] and, when paired with a wild shrike, their breeding success is comparable to pairs containing two wild birds [11]. The captive breeding program plays a crucial role in preventing this shrike subspecies from being extirpated from its Canadian range [2, 4]. The release of nearly 80 fledglings annually into the wild has stabilized the wild population of LOSH in Ontario at between 22–24 breeding pairs [2].

Despite its successes, the program experienced significant unexplained mortality among fledglings beginning in 2007. Postmortem examination of mortalities at the Toronto Zoo and the Canadian Cooperative Wildlife Health Centre (now Canadian Wildlife Health Cooperative (CWHC)) revealed generally non-specific findings, the most common being terminal gastric hemorrhage, which usually is associated with stress. The problem persisted in subsequent breeding seasons and in late 2010, the collaborators on this paper came together to investigate this threat to the recovery plan.

The purpose of this study was to 1) describe fledgling mortality trends in the captive LOSH population between 2006–2011, and 2) identify factors associated with fledgling survival. The overall goal was to identify steps to potentially mitigate fledgling mortality in the captive LOSH population.

All required research permits were obtained for this work (#POS 111, #CA 0129 and Banding #10809), and research ethics were approved by Environment Canada, Canadian Wildlife Service (National Wildlife Research Centre (NWRC)/CWS Animal Care Committee Approval, Project #RS01).

## Results

**Fig. 1:**
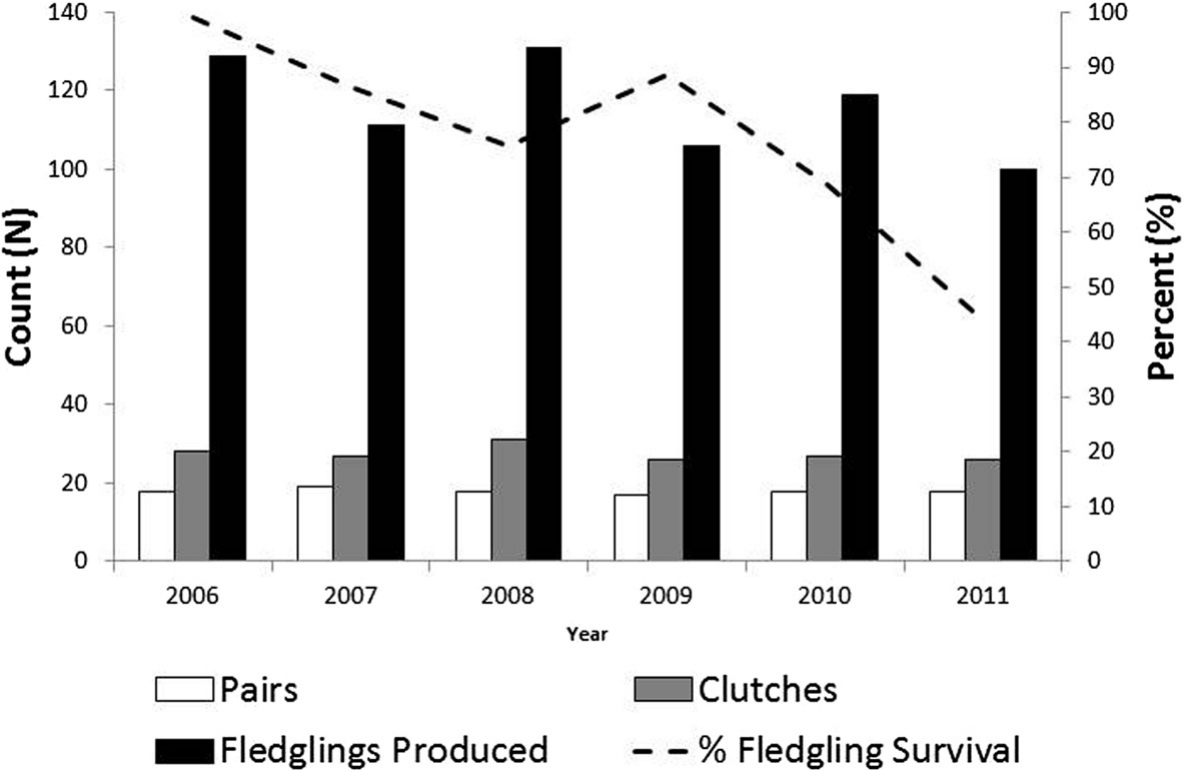
Number of breeding pairs, clutches and fledglings produced and percent survival of fledglings at Carden and Dyer’s Bay (2006–2011)

In total, between 2006 and 2011, 696 LOSH were fledged at Carden (n = 351) and at Dyer’s Bay (n = 345) (Table 1). Among these fledglings, 474 (68 %) were released, 69 (10 %) were retained in the breeding population, and 153 (22 %) died. Over the study period, on average, 79 fledglings were released annually from these two sites, ranging from a high of 111 in 2006 to a low of 21 in 2011. The proportion of fledglings that survived declined from 99 % (128/129) in 2006 to 44 % (44/100) in 2011 (Fig. 1). Among those fledglings that died, a cause of death was rarely determined. In total, 130 fledglings were submitted for necropsy during the study period from Carden and Dyer’s Bay, 64 (49 %) died of unknown causes, 20 (15 %) died of infectious causes (including bronchopneumonia, aspergillosis, capillariasis and other infections), and 11 (8 %) died from various traumatic injuries.

**Table 1:** Breeding and fledgling data – Carden and Dyer’s Bay 2006-2011

			2006	2007	2008	2009	2010	2011	Total
Carden	Breeding pairs	N	9	9	9	12	9	10	58
	Clutches	N	15	11	15	19	15	14	89
	Total fledglings produced	N	62	45	53	79	59	53	351
	Fledglings released	N (%)	62 (100)	37 (82)	41 (77)	69 (87)	30 (51)	5 (9)	244 (70)
	Fledglings retained	N (%)	0	0	3 (6)	3 (4)	3 (5)	9 (17)	18 (5)
	Fledglings dead	N (%)	0	8 (18)	9 (17)	7 (9)	26 (44)	39 (74)	89 (25)
Dyer’s Bay	Breeding pairs	N	9	10	9	5	9	8	50
	Clutches	N	13	16	16	7	12	12	76
	Total fledglings produced	N	67	66	78	27	60	47	345
	Fledglings released	N (%)	49 (73)	54 (82)	48 (62)	20 (74)	43 (72)	16 (34)	230 (67)
	Fledglings retained	N (%)	17 (25)	5 (8)	7 (9)	2 (7)	6 (10)	14 (30)	51 (15)
	Fledglings dead	N (%)	1 (1)	7 (11)	23 (29)	5 (19)	11 (18)	17 (36)	64 (19)

The following variables were statistically significant based on univariable analysis: study year, clutch number, number of nestlings in the nest, number of fledglings in the brood, the natural log of the breeding female age and its quadratic term (Table 2 and Table 3). The age of the breeding male was also considered for inclusion in the multivariable model based on a liberal p-value. Although statistically significant, the number of nestlings (as opposed to fledglings) was not considered for inclusion in the final model since it was highly correlated with the number of fledglings (Pearson correlation coefficient = 0.87), and the number of fledglings was more reliably assessed in the field by the staff of the breeding program.

**Table 2:** Name and description of variables included in uni-variable and multi-variable multi-level logistic regression models

Variable name	Description
Bird ID	Unique ID number for each bird in the population
Clutch ID	Unique ID number for the clutch
Pair ID	Unique ID number for the parent pair
Year	Breeding year
Breeding Location	Breeding site (Carden or Dyer’s Bay)
Cage Type	Whether the cage had 2 or 3 sections
Female breeding age	Age of female parent (years)
Male breeding age	Age of male parent (years)
Female birth year	Year the female parent was born
Male birth year	Year the male parent was born
Female breeding outcome	The breeding outcome of the female parent in the previous year (second clutch, first clutch, failed to breed, eggs hatched but no fledglings, or unpaired)
Clutch number	First or second clutch
Number of fledglings	Number of young that fledged from a unique brood
Survival	Whether or not the individual fledgling survived to be released or retained in the captive population

**Table 3:** Univariable multi-level logistic regression models* estimating the associations between breeding pair and clutch characteristics, breeding site, year, and cage type and the odds of survival among Eastern Loggerhead Shrike fledglings in a captive breeding program in Ontario (2006–2011)

Variable:	OR	95 % CI	P-value
**Site:**			
Carden	ref.		
Dyer's Bay	1.86	0.49-7.15	0.364
**Year of study (1–6):**	0.36	0.24-0.53	<0.001
**Clutch number:**			
First clutch	ref.		
2nd clutch	0.20	0.07-0.55	0.002
**Cage type:**			
Double	ref.		
Triple	1.60	0.25-10.51	0.622
**No. of fledglings in the brood**	2.54	1.68-3.85	<0.001
**No. of nestlings in the nest**	2.07	1.38-3.09	<0.001
**Birth year of breeding female**	0.83	0.66-1.05	0.119
**Birth year of breeding male**	0.85	0.68-1.07	0.179
**Breeding female age (years):**			
**log(female’s age)**	27.49	0.98-767.55	0.051
**(log(female’s age))** ^**2**^	0.19	0.05-0.74	0.017
**Breeding male age (years)**	0.80	0.63-1.02	0.068
**Breeding outcome of female in previous year:**			0.397^†^
Double-clutched	ref.		
Failed to breed	0.18	0.02-1.72	0.135
Hatched the prior year	0.43	0.05-3.68	0.443
Single clutch in year prior	1.16	0.30-4.45	0.832
Unpaired	7.54	0.09-654.40	0.375

*Univariable multi-level models include one fixed effect and random intercepts for clutch (Clutch ID) and breeding pair (Pair ID).

^†^P-value from a Wald chi-square examining the statistical significance of the entire categorical variable.

ref. = Referent category.

**Fig. 2:**
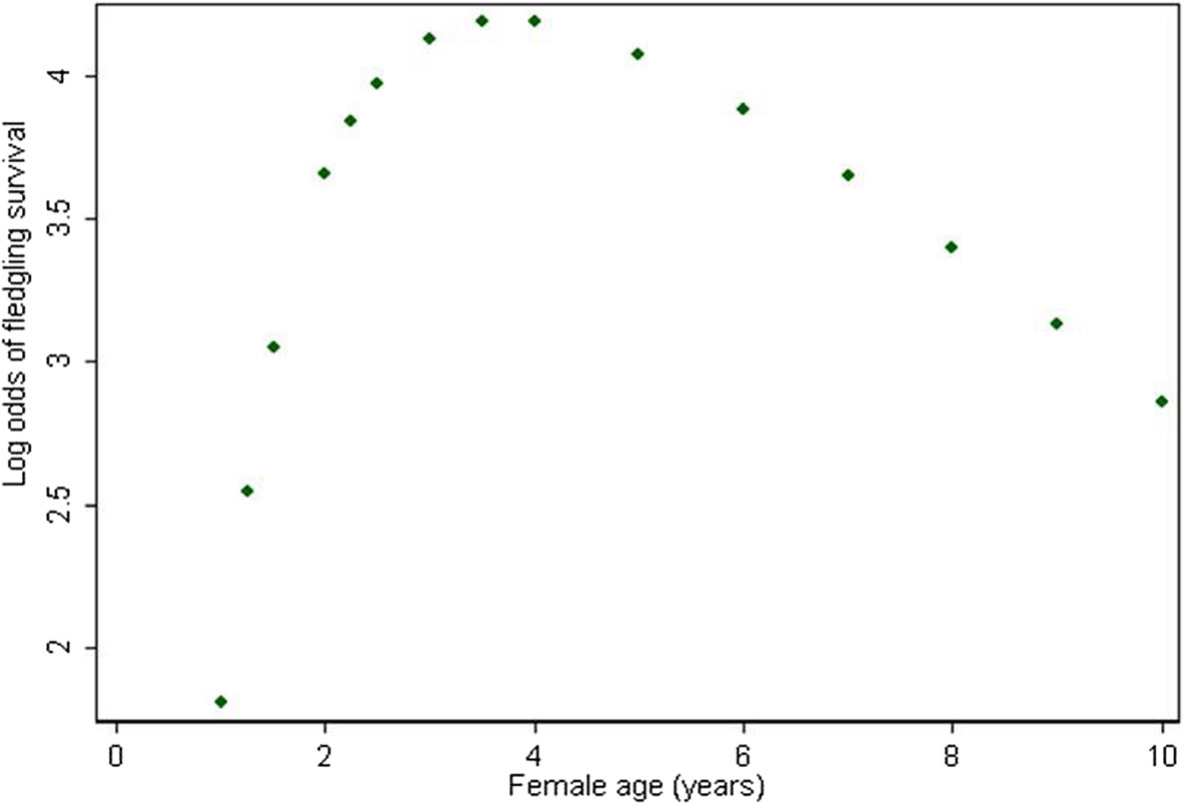
The predicted effect of breeding female age on the log odds of survival among Eastern Loggerhead Shrike fledglings in a captive breeding program in Ontario (2006–2011)

In the final multivariable model, the following variables were statistically significant: study year, clutch number, number of fledglings, and the natural log of the breeding female age and its quadratic term (Table 4). The odds of a fledgling surviving showed a significant negative (declining) trend between 2006 and 2011. The odds of surviving were significantly lower if a fledgling was part of a second clutch. As the number of fledglings increased in a clutch, there was a significantly increased odds of a fledgling surviving. As a breeding female aged, there was a marked increase in the odds of a chick surviving, but this began to decline after the female was 4 years of age (Table 4 and Fig. 2).

**Table 4:** Multivariable multi-level logistic regression model* estimating the associations between year, clutch number, fledglings in the brood, and hen’s age and the odds of survival among Eastern Loggerhead Shrike fledglings in a captive breeding program in Ontario (2006–2011)

Variable:	OR	95 % CI	P-value
**Year of study (1–6):**	0.40	0.29-0.56	<0.001
**Clutch number:**			
First clutch	ref.		
2nd clutch	0.34	0.14-0.83	0.018
**No. of fledglings in the brood:**	1.74	1.20-2.54	0.004
**Breeding female age (years):**			
log(female's age)	37.00	3.04-451.01	0.005
(log(female's age))^2^	0.25	0.09-0.70	0.008
**Random effects parameters:**	**Variance**	**95 % CI**	<0.001^†^
**Pair ID**	1.40	0.49-4.03	
**Clutch ID**	2.23	0.93-5.36	

*Multilevel model includes random intercepts for clutch (Clutch ID) and breeding pair (Pair ID).

^†^Likelihood ratio test testing the significance of the random intercepts in the model.

ref. = Referent category.

Based on the variance components, we estimated that 47.5 %, 32.2 %, and 20.2 % of the variance in survival was explained at the fledgling, clutch, and breeding pair levels, respectively, after accounting for the fixed effects in the model. Based on an intercept only model (i.e., without the fixed effects), 26.3 %, 39.6 %, and 34.1 % of the variance in survival was explained at the fledgling, clutch, and breeding pair levels, respectively.

The best linear unbiased predictors (BLUPS) at the pair-level met all the model assumptions. The BLUPS examined at the clutch-level showed homogeneity of variance, but they appeared to be left skewed. However, the inclusion of this random effect improved the fit of the model based on lower Akaike's information criteria (AIC) (489.12 vs. 504.12) and Schwarz's Bayesian information criteria (BIC) (525.33 vs. 535.80) values when the full model was compared to a model that did include the random intercept for clutch. Three observations had large Pearson residuals, but there was no evidence of recording errors, and the observations were retained in the model.

## Discussion

Our analyses suggest that clutch number, number of fledglings in the brood, and breeding female age were significant predictors of fledgling survival. Management of the captive breeding population can influence many of these factors.

Based on our multivariable model, it appears that fledgling survival is lower among second clutch birds. We hypothesize that this may be due to higher breeding stress and reduced fitness of the parents after having already fledged one brood. Lower fledgling survival arising from second clutches also may be associated with changing environmental conditions later in summer. Second clutches are uncommon among wild shrike pairs [2].

Additional analyses are needed to determine whether double clutching should be continued. It certainly increases the number of birds released into the wild, and as that is the goal of the captive breeding program then it may be appropriate to continue. However, as fewer fledglings from second clutches survive, the overall proportion of young birds that survive decreases. Consequently, the parents invest more energy per bird released in production of the second clutch birds than those from first clutches. Those energetic costs may impact reproductive success in subsequent years. Research with Eastern Kingbirds (*Tyrannus tyrannus*) demonstrated that females that fledged more young in the previous breeding year experienced lower fledgling survival than those that had fledged fewer birds the year before [12]. To investigate whether or not the practice of double clutching negatively affected the success of the female in the subsequent breeding year, we included breeding outcome for breeding female in the previous year as a variable in the univariable model; there was no association with survival. The lack of such an association may reflect the true state of nature or a lack of power in the study, due to the small size of the captive population. Because the focus of this study was to investigate mortality in a small defined population of captive birds, no sample size calculations were performed as might be done prior to a pre-planned observational study or randomized trial.

The statistical model also revealed a significant association between fledgling survival and the age of the female at breeding (Table 4 and Fig. 2). Survival among fledglings very quickly increases as the female ages up to 4 years, and then slowly decreases over time. This association may be attributed to improved breeding experience in the early years, followed by declining physiological reproductive capacity as the female ages. Alternatively, this association may be caused by a survival bias, in that successful females are more likely to be re-bred year after year. There was no equivalent association between male breeding age and fledgling survival (Table 2). Neither was there as association between the female or male birth year and fledgling survival, suggesting that there was no cohort effect (i.e., that the year of the parent birth did not affect survival of their young).

Fledgling survival was higher in broods with more fledglings. This measure may be an indicator of the overall health of the brood. For example, a greater number of fledglings could be associated with less infectious and non-infectious disease among the fledglings and/or that the parents are less stressed and better at caring for their young right through the nestling and fledgling stages. To date, infectious disease has not been identified as a significant contributing factor to juvenile mortality in the captive Ontario population of LOSH.

Other possible explanations for poor captive fledgling survival may include differences in husbandry and management between breeding sites and between years. Managers of the breeding facilities suggested that the size of cage (2 sections or 3 sections) affected survival, with higher survival in clutches raised in large (3-section) cages. However, we detected no significant difference in fledgling survival between the cage types. Similarly, there was no difference in fledgling survival between the two major breeding sites (Carden and Dyer’s Bay) during the study period, which likely reflects the similarity in management, as both sites adhered to the WPC protocol and WPC trained all the staff.

In the model presented here, we have included only data about fledgling survival. Data are available about nestling survival, but more uncertainty surrounds these estimates. Since nestlings can be challenging to count in the nest and can quickly disappear from the environment if they die, we limited our investigation to fledgling survival. However, some juvenile birds do die before fledging, and death may be associated with the same spectrum of risk factors.

In 2012, due primarily to funding constraints, the captive breeding program was restricted to one site and the density of breeding birds was substantially reduced; 32 fledglings were produced: 24 (75 %) were released, 5 (16 %) were retained, and 3 (9 %) died. The size of the release group was kept relatively large (~9-10 fledglings), as previous work revealed that returning birds were more likely to have been released in large groups of juveniles [11]. In 2013, 5 fledglings from the 2012 hatch year returned to Ontario (Personal communication, J Steiner). The period 2012–2014 has been marked by higher survival rates, and an increasing number of birds released; in 2014 nearly 100 young were released, as was the case in 2006, before issues with increasing fledgling mortality began. The reduction in breeding pair density may have reduced stress on the birds. In addition, supplements (additional vitamin E and probiotics) have been added to the diets fed in captivity. Ultimately, the goal of the program is to introduce more potential breeding birds to the wild. Consequently, examining the costs and benefits of altering breeding density and breeding intensity (e.g., 1 vs. 2 clutches) needs to be considered for the long-term success of this program.

## Conclusions

LOSH remain endangered in Canada and the captive breeding program is an important part of maintaining this population. Based on our analyses, clutch number, number of fledglings in a brood, and breeding female age were significant predictors of fledgling survival. Our research found that fledgling survival was reduced for birds from second clutches, but encouraging pairs to have a second clutch does result in a greater number of birds being released; it remains unclear if compromising survival rates to ultimately release more birds into the wild is a worthwhile compromise for increasing populations of LOSH in the wild. Long–term management decisions for the captive breeding program need to ultimately be focused on increasing the number of breeding pairs in the wild and sustaining a genetically diverse population in captivity until LOSH numbers are adequate to sustain a wild population.

## Methods

The LOSH captive breeding program in Ontario is a collaborative effort involving WPC, CWS, Toronto Zoo, African Lion Safari, Mountsberg Raptor Center, and CWHC.

Data about fledgling deaths prior to 2006 often were incomplete, and the management data captured at the various breeding sites was inconsistent. Since data collected beginning in 2006, the year before abnormal mortality began, were more complete, the study encompassed 2006–2011, during which period management had remained more-or-less consistent, even in the face of ongoing excess mortality.

Hardcopy and electronic files containing health and management information (e.g., breeding records, nutrition, health status, and disease treatment and prevention practices) were collected from WPC and the Toronto Zoo. Hardcopy data were transcribed into electronic files. Post mortem diagnostic findings were extracted from the CWHC database and from the electronic Toronto Zoo pathology files stored at the University of Guelph.

Once the available data were converted to electronic files, organised and validated, descriptive statistics (including summary counts, proportions and mean values stratified by breeding location and year) were performed to characterize the mortality observed within the captive LOSH population. Multi-level logistic regression models were subsequently constructed to identify significant variables associated with fledgling survival. A multi-level approach was selected to account for clustering by clutch and parent pair. A causal diagram was used to identify key variables to include in the model building process (Table 2). All analyses presented were carried out using Excel 2007 (Microsoft) and STATA 12 MP (StataCorp, College Station, Texas). Prior to model building, the assumption of linearity for continuous independent variables was examined graphically using locally weighted regression (i.e., lowess curves) to determine the relationship between the log odds of the outcome and each continuous explanatory variable. If the relationship was not linear the following options were considered: 1) transforming the independent variable to achieve linearity; 2) including the main effect and its quadratic term together in the model if a quadratic relationship was evident from the lowess curve; and 3) categorize the variable into quartiles if the previous options were not appropriate or could not achieve linearity. Quadratic terms were only kept in the subsequent models if they were statistically significant at the 5 % level (i.e., α = 0.05).

To avoid issues associated with collinearity during multivariable modeling, we examined correlations between independent variables using Pearson’s and Spearman's rank correlations depending on the nature of the variables (i.e., continuous vs. categorical). If the absolute correlation between two variables exceeded 70 %, only the variable that was statistically significant and/or more biologically plausible was considered for inclusion in the final multivariable model.

### i. Univariable models:

Using multi-level logistic regression models that included random intercepts for pair and clutch, built using adaptive quadrature with the “xtmelogit” command, we estimated the associations between the following independent variables and the odds of fledgling survival: breeding site (Carden vs. Dyer’s Bay), year of study, clutch number (1^st^ vs. 2^nd^), cage type (double vs. triple), number of nestlings in the nest, number of fledglings in the brood, year of birth of breeding female and male, age of breeding female and male, and breeding outcome for the female in the previous year (Table 2).

### ii. Multivariable model:

All variables that were statistically significant based on a “liberal p-value” (i.e., α = 0.20), were considered for inclusion in the multivariable multi-level logistic regression model except where two variables were highly correlated [13]. Variables were kept in the final model if they were statistically significant (α = 0.05), acted as a confounding variable, or were part of a statistically significant interaction term. A confounder was defined as a non-intervening variable whose removal from the model resulted in a 20 % or greater change in the coefficient of another statistically significant variable [13]. We examined all potential interactions among main effects that were being retained in the final multivariable model individually. If an interaction effect was statistically significant it was retained in the final model. Based on an a priori decision, we examined the potential confounding effect of site and year and the interaction between these variables regardless of their statistical significance. The variance partition coefficient for each level of the model (i.e., fledgling, clutch, and breeding pair) was estimated using the latent variable technique [13].

To assess the fit of the model, we examined the assumptions of normality and homogeneity of variance for the BLUPS graphically. If the BLUPS did not meet these model assumptions, we compared models with and without the random intercept(s) using AIC and BIC to confirm that the addition of these terms improved the model fit. We also examined Pearson residuals to determine if there were any outlying observations. Observations with Pearson residuals with an absolute value greater than 3 were examined for potential recording errors.

## Abbreviations

LOSH: Loggerhead Shrike, Eastern subspecies (*Lanius ludovicianus* ssp.)
COSEWIC: Committee on the Status of Endangered Wildlife in Canada
SARA: Species at Risk Act
MBCA: Migratory Birds Convention Act
CWS: Canadian Wildlife Service
NWRC: National Wildlife Research Centre
WPC: Wildlife Preservation Canada
CWHC: Canadian Wildlife Health Cooperative
BLUPS: best linear unbiased predictors
AIC: Akaike’s information criteria
BIC: (Schwarz’s) Bayesian information criteria
